# Freshwater Sediment Microbial Communities Are Not Resilient to Disturbance From Agricultural Land Runoff

**DOI:** 10.3389/fmicb.2020.539921

**Published:** 2020-10-15

**Authors:** Rachelle E. Beattie, Aditya Bandla, Sanjay Swarup, Krassimira R. Hristova

**Affiliations:** ^1^Department of Biological Sciences, Marquette University, Milwaukee, WI, United States; ^2^Singapore Centre for Environmental Life Sciences Engineering, Nanyang Technological University, Singapore, Singapore; ^3^NUS Environmental Research Institute, National University of Singapore, Singapore, Singapore; ^4^Department of Biological Science, National University of Singapore, Singapore, Singapore

**Keywords:** resistance, resilience, microbial community structure, freshwater, agricultural runoff

## Abstract

Microorganisms are critically important for the function of surface water ecosystems but are frequently subjected to anthropogenic disturbances at either acute (pulse) or long-term (press) scales. Response and recovery of microbial community composition and function following pulse disturbance is well-studied in controlled, laboratory scale experiments but is less well-understood in natural environments undergoing continual press disturbance. The objectives of this study were to determine the drivers of sediment microbial compositional and functional changes in freshwaters receiving continual press disturbance from agricultural land runoff and to evaluate the ability of the native microbial community to resist disturbance related changes as a proxy for freshwater ecosystem health. Freshwater sediments were collected seasonally over 1 year in Kewaunee County, Wisconsin, a region impacted by concentrated dairy cattle farming, manure fertilization, and associated agricultural runoff which together serve as a press disturbance. Using 16S rRNA gene amplicon sequencing, we found that sediments in locations strongly impacted by intensive agriculture contain significantly higher abundances (*p* < 0.01) of the genera *Thiobacillus, Methylotenera, Crenotrhix, Nitrospira*, and *Rhodoferax* compared to reference sediments, and functions including nitrate reduction, nitrite reduction, and nitrogen respiration are significantly higher (*p* < 0.05) at locations in close proximity to large farms. Nine species-level potential human pathogens were identified in riverine sediments including *Acinetobacer lwoffi* and *Arcobacter skirrowii*, two pathogens associated with the cattle microbiome. Microbial community composition at locations in close proximity to intensive agriculture was not resistant nor resilient to agricultural runoff disturbance within 5 months post-disturbance but did reach a new, stable microbial composition. From this data, we conclude that sediment microbial community composition is sensitive and shifts in response to chemical and microbial pollution from intensive agriculture, has a low capacity to resist infiltration by non-native, harmful bacteria and, overall, the natural buffering capacity of freshwater ecosystems is unable to fully resist the impacts from agricultural press disturbance.

## Introduction

Freshwater ecosystems are a vital natural resource, providing sources of drinking water, food, animal habitats, and recreation. Sediment microbial communities within freshwater ecosystems support important functions including carbon and nitrogen cycling due to their high abundance, diversity, and stability (Nealson, 1997). Sediments also serve as the primary site of accumulation and attachment of both bacteria and chemical pollutants; thus, sediment microorganisms are exposed to a wide variety of anthropogenic pollutants (Noe and Hupp, 2005; Devarajan et al., 2015; Saxena et al., 2015). In ecological systems, pollution exposure events are termed “disturbances.” Disturbance events are classified by their duration of impact as either pulse (short-term, acute) or press (long-term, continuous) disturbance (Shade et al., 2012a). Disturbance is a primary factor in microbial community structure and diversity (Shade et al., 2012a,b), and microbial community stability within freshwater sediments is crucial for maintaining ecosystem functioning. The impact of disturbance on microbial ecosystems can be measured by two factors: resistance, or the degree to which a community can withstand and remain unchanged by disturbance, and resilience, or the rate at which a community returns to a pre-disturbance composition (Allison and Martiny, 2008; Shade et al., 2012a). Measuring changes in microbial community composition following anthropogenic disturbance helps predict ecosystem functioning and health (Galand et al., 2016; Santillan et al., 2019).

Agriculture is the primary source of non-point source (NPS) pollution and degraded water quality in rivers and streams (USEPA, 2017). Livestock and field crop farming practices contribute to both reduced water quality and ecosystem functioning by introducing nitrate, phosphorus, heavy metals, antibiotics, and non-native microorganisms into waterways via agricultural land runoff (Pruden et al., 2013; Manyi-Loh et al., 2016; USEPA, 2017; Le et al., 2018). Microbial pathogens originating from manure are a primary concern. Livestock manure is known to contain pathogens that cause infection in humans including members of the genera *Arcobacter* and *Acinetobacter* and more well-known pathogens including methicillin resistant *Staphylococcus aureus* and toxic *Escherichia coli* (Igbinosa et al., 2012; Giacometti et al., 2015; Guimarães et al., 2017; Klotz et al., 2017). Input of high concentrations of nitrate and phosphorus from agricultural fertilization runoff can also alter sediment microbial community composition. Henson et al. (2018) found that members of the family *Comamondaceae* and genera *Mucilaginibacter, Pseudospirillum, and Novosphingobium* strongly correlate with nitrate concentrations while members of the class Holophagaceae, family *Gemmatimonadaceae*, and genus *Nitrospira* strongly correlate with phosphate concentrations. Additionally, it has been well-documented that increases in *Cyanobacteria*, particularly genera *Microcystis, Anabaena, Planktothrix*, and *Aphanizomenon*, are associated with freshwater eutrophication events (Havens, 2008; Dolman et al., 2012; Graham et al., 2012). The responses of microbial communities to disturbance are dependent on multiple interrelated factors including the type, number, length, and severity of disturbance (Reinold et al., 2019). Several studies report high sensitivity of microbial communities to anthropogenic disturbances and the Microbiome Stress Project (Rocca et al., 2019) provides a comprehensive database facilitating comparisons of major findings. However, the resilience of native microbial communities following disturbance in the environment is still largely unexplored and most reports are based on controlled laboratory experiments (Mooshammer et al., 2017; Lourenço et al., 2018). Understanding of the impact of press manure disturbance on the resistance and resilience of microbial community composition is necessary to evaluate ecological and human health risks, as resistant and resilient microbial communities tend to be more diverse, functionally redundant, and better able to resist influxes of pathogenic organisms and chemical pollutants (Allison and Martiny, 2008; Shade et al., 2012a).

In our study, the primary research question is: how does agricultural land runoff impact microbial community composition and function in freshwater sediments in a region of intensive agriculture that suffers from reduced freshwater quality? We have addressed this question in Kewaunee County, Wisconsin, which is home to 16 Confined Animal Feeding Operations (CAFOs, i.e., operations exceeding 1,000 animal units) and more than 160 smaller farms. Each year, approximately 151,000 tons of manure solids and 784 million gallons of liquified manure are applied onto land in the county between the mandated spreading dates of April 15–December 31 (KCDL and WC, 2019), serving as a repeated, press disturbance. Due to subsequent manure runoff, local river watersheds remain at high risk for repeated NPS pollution and are listed as impaired waterways by the US-EPA (KCDL and WC, 2019). Additionally, the region has a shallow, fractured karst bedrock geology that contributes to surface-to-groundwater pollution of drinking water wells with contaminants including nitrate, bacteria, and viruses (Borchardt et al., 2019).

We hypothesized that (1) sediment microbial communities in the highly impacted Kewaunee River watershed will be resilient to press disturbance from manure fertilization runoff due to long-term stress and adaptation to pollutants in the region; (2) microbial community composition will shift seasonally during the manure fertilization period toward communities enriched with copiotrophs (such as members of the phylum *Cyanobacteria* and members of the genera *Pseudomonas* and *Novosphingobium*); and (3) potential pathogens associated with the cattle microbiome will be detected more frequently in freshwater sediments at river locations impacted by manure runoff from intensive agriculture. We tested these hypotheses using 16S rRNA gene sequencing combined with exact sequence matching and functional inferences.

## Materials and Methods

### Sediment Sampling

**FIGURE 1:**
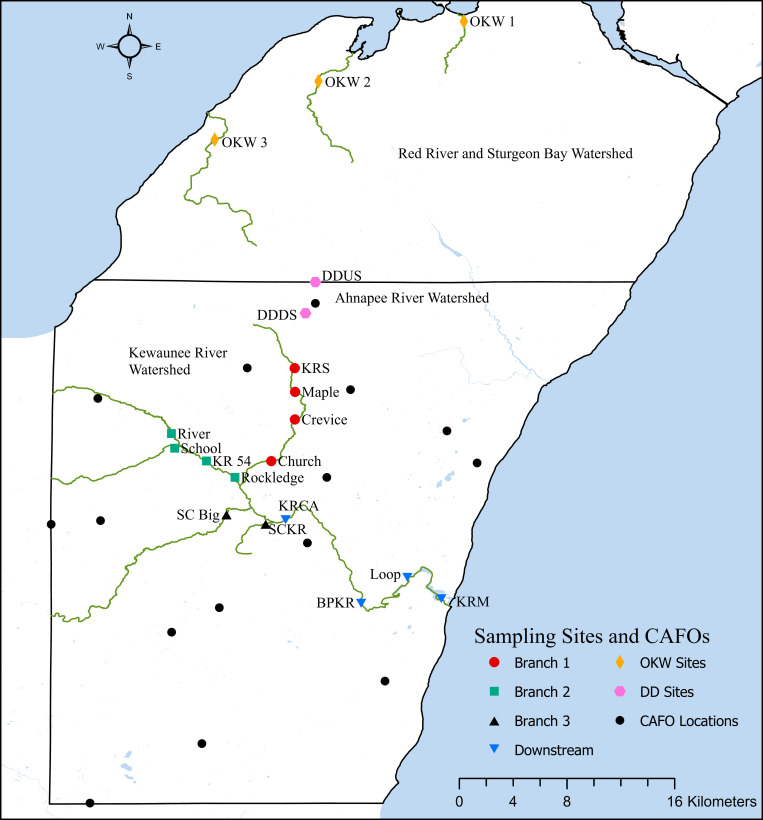
Sampling locations, CAFO locations, and river watershed locations in Kewaunee and Door counties, Wisconsin.

The three major watersheds within Kewaunee County, Wisconsin have been fully described elsewhere (Beattie et al., 2018). Briefly, Kewaunee County contains three primary river watersheds, which encompassed 15 CAFO farms at the time of this study. This work focuses primarily on locations within the Kewaunee River watershed due to the high impact of CAFO farms, manure fertilized cropland, and associated runoff on the Kewaunee River surface water ecosystem; however, we collected additional samples immediately north and south of a CAFO farm in northern Kewaunee County and samples outside of the impact of CAFOs in adjacent Door County, Wisconsin. Grab sediment samples were collected from the riverbanks of seasonally accessible sites at a total of 19 riverine locations in Kewaunee and Door Counties, Wisconsin in February, May, September, and October 2017. Fourteen sample sites fall within the Kewaunee River watershed, two sample sites fall within the Ahnapee River watershed immediately north and south of a CAFO farm, and three sites fall within the Red River and Sturgeon Bay watershed which receives no impact from CAFO farming (Beattie et al., 2018) (Supplementary Table S1 and Figure 1). These collection timepoints were chosen to capture sediment microbial communities before the manure fertilization period begins in Kewaunee County (pre-disturbance, February), during the manure fertilization period (disturbance, May), and after the predominant manure fertilization period (post-disturbance, September and October). Sediments at these sites are classified as silty-sandy and because sediments were collected from the riverbank, no significant differences in flow rate were found regardless of sampling location or season. At each site on each date, three grab samples totaling approximately 1 kg were collected from the top 10 cm of sediment within a 1 m^2^ area. Sediment samples were homogenized in the field, transported on ice, stored at 4°C, processed in the lab within 48 h, and subsamples were kept frozen at −20°C. Additionally, one composite manure sample (multiple cattle, fully homogenized before sampling) from a dairy CAFO farm was collected in May 2017 and two composite manure samples from a < 200 head beef farm were collected in May and September 2017 for reference. A total of 68 sediment samples were collected for this study.

### DNA Extraction

Sediment and manure were subsampled in the laboratory for a total of two separate DNA extractions. DNA was extracted from 0.5 g of sediment or manure using the DNeasy PowerSoil Kit (Qiagen) according to manufacturer instructions. Duplicate DNA extractions were pooled for downstream analyses.

### DNA Sequencing and Amplicon Sequence Mapping

Universal primers were used to amplify the V3–V4 hypervariable region of the 16S rRNA gene followed by next generation sequencing (NGS) (McGovern et al., 2018). Paired-end sequencing of sediment and manure DNA was performed by the University of Wisconsin Biotechnology Center (Madison, WI, United States) with the Illumina MiSeq Platform. Primer sequences were removed from the raw demultiplexed reads using cutadapt v1.18. Reads were subsequently processed using DADA2 v1.9 (Callahan et al., 2016). Sequences containing ambiguous bases and phiX sequences were removed and only those reads with a minimum base quality score of two being retained for subsequent steps. Forward and reverse reads were then truncated to 250 and 230 bp, respectively, and were only retained if maximum expected errors associated with such reads were equal to or less than three (forward) or four (reverse) respectively. Reads were error-corrected and pooled prior to inferring sample composition. Error-corrected reads were then merged to yield the complete sequence spanning the sequenced hypervariable region. Chimeric sequences were subsequently removed prior to taxonomic annotation. Taxonomy was assigned using the RDP classifier based on the SILVA database v132. Species-level identification was accomplished using exact string-matches against a customized variant of the same database (Callahan, 2018). The resulting amplicon sequence variant (ASV) table was further processed by removing any ASVs with less than five reads across all samples and/or ASVs present in less than two samples. Raw sequence data were submitted to the NCBI Sequence Read Archive (SRA) under accession: PRJNA555250.

Potential pathogens were identified at the species level using the DADA2 extension for 100% exact sequence matching of sample sequences to sequenced reference strains within the Silva database (Callahan, 2018). To identify potential pathogens within each sample, the taxonomy assignment output file for all ASVs was first subset to 146 genera known to contain pathogens curated by previous studies (Li et al., 2015; Subirats et al., 2017; Fang et al., 2018). Next, those genera were subset again to only those ASVs mapped to species level using the strict 100% sequence matching criteria. Last, potential pathogens from each sample were identified from known human pathogenic species curated in the above studies followed by a confirmatory literature search of human infection cases with identified pathogenic species.

### Pollution Indicator Measurements

Field measurements included dissolved oxygen, pH, and water temperature (Mettler Toledo pH/ion Meter), and a portion of these results have been reported in previous work (Beattie et al., 2018). Agricultural pollution indicators nitrate (^${\mathrm{NO}}_{3}^{–}$)^, phosphate (^${\mathrm{PO}}_{4}^{3–}$^), total coliform bacteria, and *E. coli* were measured in river water from sediment collection sites. Nitrate and phosphate were measured using Ion Chromatography in unaltered water samples (Dionex ICS-1100, Thermo Scientific). Total coliform bacteria and *E. coli* were measured in 100 mL of river water within 24 h of collection using the USEPA approved Colilert Quanti-Tray/2000 Method (United States Environmental Protection Agency, 2010). Metal concentrations within sediments were measured using Inductively Coupled Plasma Mass Spectrometry (ICP-MS) following digestion with concentrated nitric acid.

### Statistical Analyses

Statistical analyses were performed in both PRIMER-E version 7 with the PERMANOVA + add on package and R version 3.5.2. The *phyloseq* package in R was used to calculate relative abundances of raw ASV counts based on taxonomic classification (McMurdie and Holmes, 2013). To determine microbial community patterns based on beta-diversity, non-metric multidimensional scaling (NMDS) plots were produced from a Bray-Curtis dissimilarity matrix of ASV relative abundance in conjunction with permuted multivariate homogeneity of dispersions (PERMDISP) and permuted multivariate analysis of variance (PERMANOVA). From these analyses we found no significant differences in microbial community composition between samples from the same river branch (Kewaunee River) or watershed; thus we combined samples by river branch or watershed for all downstream analyses as Branch 1, Branch 2, Branch 3, and Downstream (Kewaunee River), DD sites (immediately north and south of a CAFO), and OKW sites (sites outside the Kewaunee River watershed in Door County); see Figure 1 and Supplementary Table S1 for full details. Additionally, distance based linear models (distLM) and distance-based redundancy analysis (dbRDA) were performed in PRIMER-E to determine agricultural pollution factors impacting microbial community composition variation (Clarke and Gorley, 2015). Functional Annotation of Prokaryotic Taxa (FAPROTAX) software was used to assign putative functional roles to ASVs (Louca et al., 2016). Briefly, FAPROTAX assigns functional roles to ASVs at the genus or species level *only if all* cultured members of that group have the assigned function. Relative functional group abundances in each sample are then calculated as the total ASVs assigned to a particular function normalized to the total number of ASVs that were assigned a functional role in a particular sample.

Resistance and resilience measurements of sediment alpha diversity (within sample) and beta-diversity (between samples) pre- and post-manure fertilization disturbance were calculated with February samples considered “pre-disturbance,” May samples considered “disturbance,” September samples considered “4 months post-disturbance,” and October samples “5 months post-disturbance.” We considered May sample” disturbance” due to the start of the fertilization period (April 15^*th*^) and the intensity of manure fertilization at this timepoint. We postulate that no significant difference in alpha or beta-diversity between pre-and post-disturbance samples indicates community resistance while a significant difference pre- and post- disturbance with a return to the pre- disturbance state indicates community resilience. Resistance and resilience indices at the alpha-diversity level were calculated using estimators of sample diversity, richness, and evenness including Chao1, ACE, Shannon, and Inverse Simpson diversity indices. Resistance and resilience at the beta-diversity level were estimated by calculating the difference in beta-diversity between pre- and post-disturbance microbial communities using both PERMANOVA (analysis of variance) and ANOSIM (analysis of similarity) tests, as in Shade et al. (2012b) and Zhang et al. (2017).

## Results and Discussion

### Microbial Community Diversity, Composition, and Function Within Sediments Are Impacted by Pollutants From Agricultural Land Runoff

The specific impact of microbial and chemical contamination from manure runoff on the stability of sediment microbial communities in freshwater ecosystems is not well-characterized. In order to determine microbial community composition in freshwater sediments both with and without impact from intensive agriculture runoff over time, we collected a total of 68 seasonal sediment samples and three representative manure samples for 16S rRNA amplicon sequencing. ASVs based on NGS of 16S rRNA genes were inferred from quality filtered reads and downstream sample processing resulting in a total of 3,545,815 sequences mapped to 16,240 distinct ASVs. To answer our central hypotheses, we first investigated if the varying levels of contamination in the sampled regions impacted the microbial community diversity, composition, and function within sediments on a spatial scale. Next, we analyzed the community composition by river location over time to determine if microbial communities are resistant or resilient to manure fertilization runoff and if spatial location plays a role in the microbial community response to this temporal press disturbance.

**FIGURE 2:**
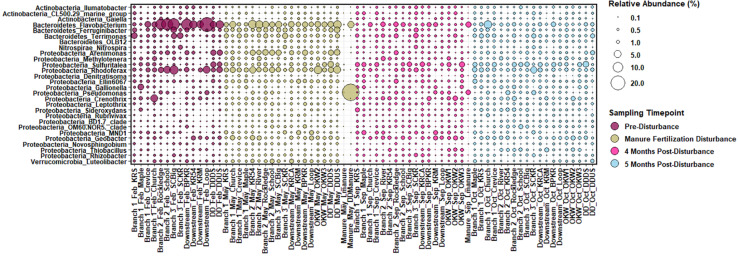
Relative abundance of sediment and manure amplicon sequence variants (ASVs) mapped to “highly abundant” genera, or those with a relative abundance of at least 0.25% across all sediment samples displayed per sample. ASVs are named by Phylum_Genus on the y-axis and sample branch and date are indicated with the site name on the x-axis.

In our samples, the core sediment microbial community (ASV taxa present in at least 50% of sediment samples) is comprised of members of the phyla *Acidobacteria, Actinobacteria, Bacteroidetes, Chloroflexi, Cyanobacteria, Proteobacteria*, and *Verrucomicrobia* among others. ASVs present in sediments above 0.25% relative abundance at the genus level were considered “highly abundant” taxa and are displayed in Figure 2. Highly abundant taxa in sample sediments include the genera *Pseudomonas, Sulfuritalea, Novosphingobium*, and *Thiobacillus* from phylum *Proteobacteria*; *Flavobacterium* from the phylum *Bacteroidetes*; *Nitrospira* from the phylum *Nitrospirae*; *Gaiella* from the phylum *Actinobacteria*, and *Luteolibacter* from the phylum *Verrucomicrobia.* Phyla *Proteobacteria, Bacteroidetes, Cyanobacteria* and *Verrucomicrobia* are commonly reported as abundant members of freshwater ecosystems, equating to more than 90% of taxa in a meta-analysis (Newton et al., 2011). Additionally, in comparison to marine and intertidal sediments, freshwater sediments are enriched in members of the genus *Nitrospira* and phylum *Verrucomicrobia* (Wang et al., 2012), both of which are highly abundant in sediments from this study. However, as Wang et al. (2012) notes, comparison between freshwater riverine sediment studies is difficult because these sediments have historically been the least studied despite their functional importance. Reference manure samples contained microbial communities that differed substantially from the sediment core community; however, manure did contain elevated abundances of the genera *Flavobacterium* and *Pseudomonas* (Figure 2).

Microbial community alpha diversity (within sample diversity) was estimated before, during and after manure fertilization disturbance using Chao1, ACE, Shannon, and Inverse Simpson diversity metrics within samples clustered by sampling location (Supplementary Figure S1). When compared within sampling location over time, the microbial community evenness and richness of samples from both highly impacted branches and low or non-impacted branches of the river did not differ significantly (*p* > 0.05). However, the diversity of microbial communities within sediment samples from highly impacted locations was significantly higher (*p* < 0.05) 4 months post-disturbance compared to their pre-disturbance state (Supplementary Figures S1C,D, Branch 2 and Downstream sites). Other studies in coastal (Galand et al., 2016) and freshwater sediments (Zhang et al., 2017) have also found that disturbance increases diversity in sediment microbial communities. Microbial communities from less or non-impacted sites were relatively stable over the course of the sampling regimen.

Spatial differences in the abundance of dominant taxa are evident from Figure 2; thus, we investigated both spatial and temporal patterns of the sediment microbial communities across the three sampled watersheds. Using Bray-Curtis dissimilarity to quantify beta diversity of sediments (between sample diversity) we compared differences in sampling watershed, sampling location, and sampling timepoint (pre- to post-disturbance) for all samples. Sediments from different watersheds contained significantly different microbial community composition (PERMANOVA, *p* = 0.001 main test, *p* < 0.01 *post hoc t*-tests; Supplementary Table S2). This suggests that spatial differences between watersheds strongly influence sediment microbial community structure likely due to the high proportion of agricultural land use (non-point source pollution) and CAFO presence (point source pollution) impacting sample locations within the Kewaunee River watershed (79% agricultural land, 6 CAFOs) compared to the Ahnapee River watershed samples (71% agricultural land and 1 CAFO) and Red River-Sturgeon Bay watershed (57% agricultural land and 0 CAFOs) (KCDL and WC, 2019). Within the Kewaunee River watershed, we found microbial communities are spatially and temporally structured with significant compositional differences present between all branches of the river but not between all months (see Figure 1 and Supplementary Table S1 for sample sites within each river branch). Pre-disturbance and manure fertilization disturbance samples are significantly different from both each other and post-disturbance samples, but samples from post-disturbance samples are not significantly different from one another (*p* = 0.83 *post hoc t*-test, Table 1). Ecological theory supports differences in microbial community composition both over time and with increased geographic distance (Nemergut et al., 2013); however, the strong spatial gradients in sediments (including gradients of agricultural pollutants) suggest that sediment microbial communities vary more strongly on spatial scales (Lozupone and Knight, 2007).

**TABLE 1:** Microbial community composition response to manure fertilization disturbance measured by differences in beta-diversity between pre- and post-disturbance samples within the Kewaunee River watershed.

ANOSIM*				
	*Before disturbance*	*Manure fertilization*	*4 months post*	*5 months post*
*Before disturbance*		**0.003**	**0.01**	**0.002**
*Manure fertilization*	0.195		**0.006**	**0.001**
*4 months post*	0.144	0.135		0.782
*5 months post*	0.253	0.193	–0.031	

*Upper triangle values represent *p*-values of the ANOSIM test (bolded values are significant) while lower triangle values represent the ANOSIM *R* statistic of differences in beta-diversity similarity between sampling dates.

**Table:** 

PERMANOVA +				
	*Before disturbance*	*Manure fertilization*	*4 months post*	*5 months post*
*Before disturbance*		**0.01**	**0.001**	**0.001**
*Manure fertilization*	1.3759		**0.018**	**0.001**
*4 months post*	1.5085	1.3924		0.83
*5 months post*	1.5830	1.621	0.85224	

*+ Upper triangle values represent *p*-values of the PERMANOVA test (bolded values are significant) while lower triangle values represent PERMANOVA pair-wise *t*-tests of differences in beta-diversity variance between sampling dates.*

**FIGURE 3:**
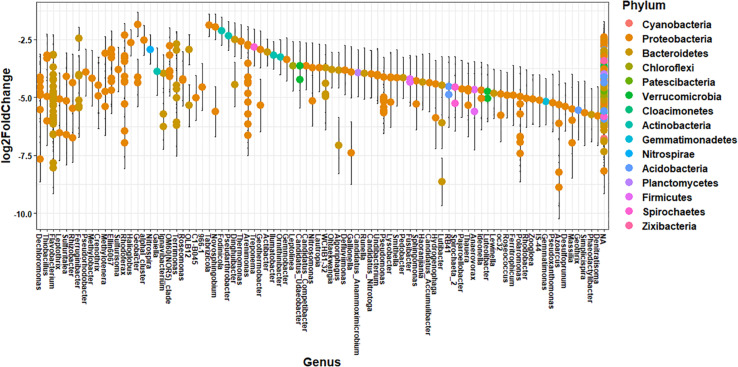
Differential abundance of ASVs between Branch 2 locations (highly impacted by intensive agriculture) and OKW locations combined (no impact from intensive agriculture) with ASVs that are significantly (*p* < 0.01, Benjamini–Hochberg correction) more abundant in Branch 2 displayed on the plot. Each point is the log2 fold change ± standard error of one ASV representing the estimate of the difference in the abundance of that ASV between Branch 2 and OKW sampling locations. Genera are listed on the x-axis and colored by phylum.

The impact of agricultural land runoff on sediment microbial community composition differs spatially due to the presence or absence of CAFOs in each watershed and differences in agricultural land use. T Thus, we investigated the specific taxonomic differences in ASV abundance between sediment samples from the branch of the Kewaunee River with the highest agricultural pollution impact (Branch 2) and the locations least impacted by agricultural pollution (OKW sites) using differential abundance analysis (Figure 3). A total of 412 ASVs from 14 named phyla and 87 named genera were significantly more abundant (adjusted *p* < 0.01) in Branch 2 sites of the Kewaunee River compared to OKW sites; the effect size estimate displayed in Figure 3 expresses the estimate of the difference in the abundance of one ASV between Branch 2 and OKW sampling locations. These include ASVs of genera *Thiobacillus*, *Methylotenera, Crenotrhix, Nitrospira*, and *Rhodoferax* among others. Of ASVs mapped to the species level, those that were more abundant in Branch 2 sites include *Arenimonas aquatica, Leptothrix cholodnii, Nitrospira defluvii, Flavobacterium flevense, Polaromonas naphthalenivorans, Zoogloea oleivorans, Flavobacterium psychrolimnae*, and *Flavobacterium xinjiangense*. Members of these genera and associated higher level taxonomy are commonly found in freshwater environments (Robertson and Kuenen, 2006; Newton et al., 2011; Kalyuzhnaya et al., 2012) but are frequently present in higher abundances when agricultural pollutants are present (Wang et al., 2016; Huang et al., 2017; Yu et al., 2017). In particular, *Nitrospira* taxa have been found linked to fertilization practices in agricultural systems (Freitag et al., 2005), and *Flavobacterium* taxa are enriched in dairy cattle manure (Pandey et al., 2018), suggesting manure fertilization practices in Kewaunee County may contribute to the elevated abundances of these microorganisms in Branch 2.

**FIGURE 4:**
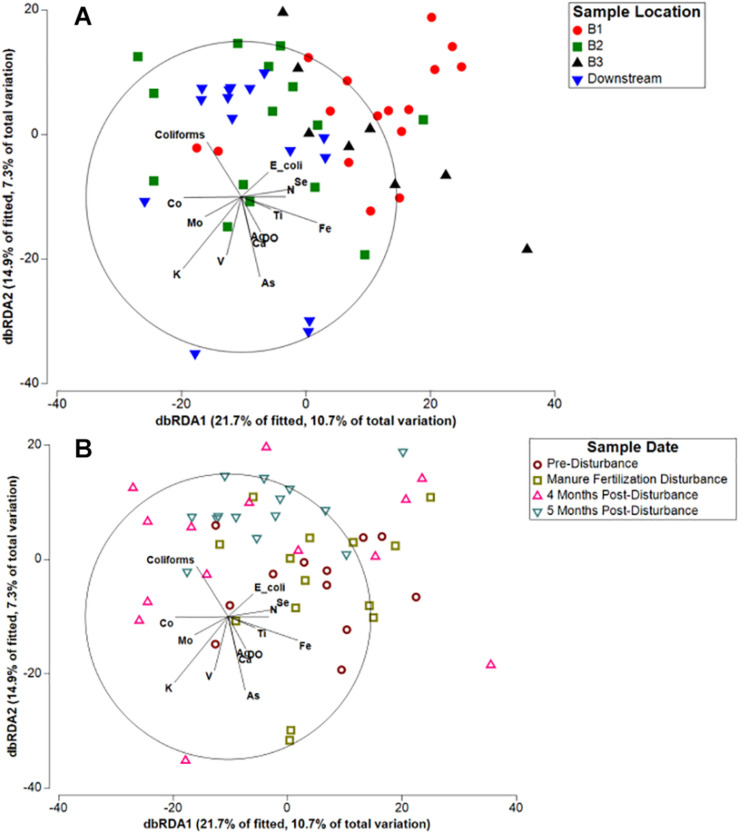
Distance-based redundancy analysis (db-RDA) of Kewaunee County watershed sediment microbial communities in response to measured environmental factors clustered by **(A)** river sampling location and **(B)** sampling timepoint. Variables found to explain variation in microbial community composition that were at or near significance (*p* < 0.1) and with moderate to strong correlations (*r*^2^ > 0.2) are displayed in the plot (for all variables in the model, see Supplementary Table S2).

To investigate how specific agricultural pollutants or environmental variables influence spatial and temporal differences in microbial community composition, we used distance-based redundancy analysis (dbRDA; Figures 4A,B). Concentrations of nitrate (N), dissolved oxygen (DO), total fecal coliforms, *E. coli*, and 12 metals previously reported (Beattie et al., 2018) significantly contributed to the variation in microbial communities (Supplementary Table S2). Multiple manure associated pollution indicators (nitrate, total fecal coliforms, heavy metals) significantly influenced the variation in sediment microbial community composition, together contributing to 56.5% of the explained variation identified by dbRDA (Figure 4A). Although microbial communities grouped by location do appear heterogeneous on the plot rather than forming tight clusters, Branch 2 and Downstream samples are closely ordinated with one another and vary strongly along dbRDA2, suggesting the spatial pattern of microbial communities is a result of the local concentration of pollutants. Spatial patterning within the Kewaunee River watershed was stronger than temporal patterning within the watershed as a whole (Figure 4B) and was consistent across all timepoints, suggesting local concentrations of pollutants strongly select for sediment microbial community composition, similar to the results of a multi-year study by Fortunato et al. (2012). Temporally, some patterning is evident as samples collected during the manure fertilization disturbance period (May) are more separated from post-disturbance samples and are strongly correlated with *E. coli* and nitrate, two indicators of fecal contamination (Figure 4B). However, the spatial patterning associated with key contaminants appears to be driving the microbial community composition of Kewaunee County sediments.

**FIGURE 5:**
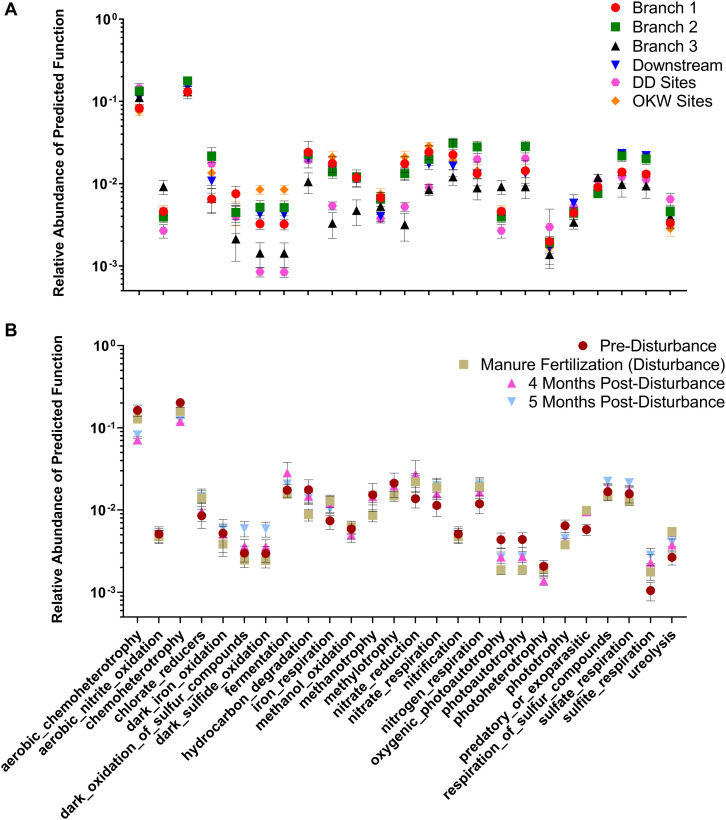
Relative abundance of functions assigned by FAPROTAX to each sample clustered by **(A)** river sampling location and **(B)** sampling timepoint. Mean relative abundance ± standard error is shown for each cluster on a log scale.

Changes in the abundance of microbial taxa within sediments can alter key ecosystem processes due to the functional importance of microorganisms; thus, putative microbial community functions were analyzed via Functional Annotation of Prokaryotic Taxa. Functional roles inferred using FAPROTAX were only assigned to 16.8% of ASVs in this study. However, generalizations about dominant functions in samples and taxa associated with those functions can be assessed. Most samples were enriched for ASVs assigned to aerobic chemoheterotrophy (Figures 5A,B), which can likely be attributed to the increased carbon available from agricultural land runoff in the sampled region as indicated by other studies (Das et al., 2017; Chen et al., 2018). Interestingly, Branch 2 and DD sites, both highly impacted by intensive agriculture, contained the highest relative abundance of chemoheterotrophy functions while OKW and Branch 1 sites, either unimpacted or less impacted by agricultural land runoff, contained the lowest relative abundance of these functions (Figure 5A). Additionally, chemoheterotrophy functions were higher during disturbance than post-disturbance, supporting our hypothesis that manure fertilization disturbance increases available carbon sources (Figure 5B). Functions assigned to the remainder of highly abundant genera in sediments across all study locations (Figure 2) can be found in Table 2.

**TABLE 2:** Functions assigned to highly abundant genera via FAPROTAX.

Genus or species	Assigned functions
*Arenimonas*	Chemoheterotrophy
*Crenothrix*	Chemoheterotrophy, hydrocarbon degredation, methanotrophy, methylotrophy
*Denitratisoma oestradiolicum*	Chemoheterotrophy, nitrate reduction, nitrate respiration, nitrogen respiration
*Ferruginibacter*	Chemoheterotrophy
*Flavobacterium*	Chemoheterotrophy
*Gallionella*	Dark iron oxidation
*Geobacter*	Iron respiration
*Leptothrix cholodnii*	Dark iron oxidation
*Methylotenera*	Chemoheterotrophy, methanol oxidation, methylotrophy
*Nitrospira*	Aerobic nitrate oxidation, nitrification
*Novosphingobium*	Chemoheterotrophy
*Pseudomonas*	Chemoheterotrophy
*Rhodoferax ferrireducens*	Chemoheterotrophy, nitrate reduction, nitrate respiration, nitrogen respiration, iron respiration
*Thiobacillus*	Dark oxidation of sulfur compounds, dark sulfide oxidation

Additional functions identified in freshwater sediments included fermentation, nitrate reduction, nitrate respiration, and sulfate respiration (Figure 5A). Functions including nitrate reduction and respiration were significantly more abundant (*p* < 0.05) in Branch 2 samples than OKW samples, indicating potential differences in microbial community function in areas highly impacted by intensive agriculture compared to unimpacted locations. This finding was also reflected by the ASV taxa mapped to these functions including *Rhodoferax* and *Nitrospira* which were found to be both more abundant in highly impacted sites (i.e., Branch 2; Table 2). *Nitrospira* are ubiquitous members of the nitrogen cycle, known to perform key functions including nitrite oxidation from ammonia and facilitation of nitrate reduction (Lücker et al., 2010), thus ammonia inputs to freshwaters from manure runoff can increase the abundance of members of this genus and increase the concentration of nitrate in surface waters (Daims et al., 2015). Higher concentrations of nitrate contribute to freshwater eutrophication and threatens human health when present in drinking water supplies. *Rhodoferax* and *Thiobacillus* taxa are known to reduce iron and oxidize sulfur, respectively (Finneran et al., 2003; Yang et al., 2010). Additionally, members of the genera *Methylotenera* and *Crenothrix* can utilize reduced carbon sources, such as methane, as their primary carbon source (Oshkin et al., 2015; Oswald et al., 2017); as such, they play a major role in freshwater carbon cycling. Together, the increased abundances of these genera at sites highly impacted by intensive agriculture likely reflects their functional importance and ability to utilize nutrient inputs from agricultural land runoff in freshwater sediments.

### Microbial Resistance and Resilience of Sediments Impacted by Agricultural Land Runoff

**FIGURE 6:**
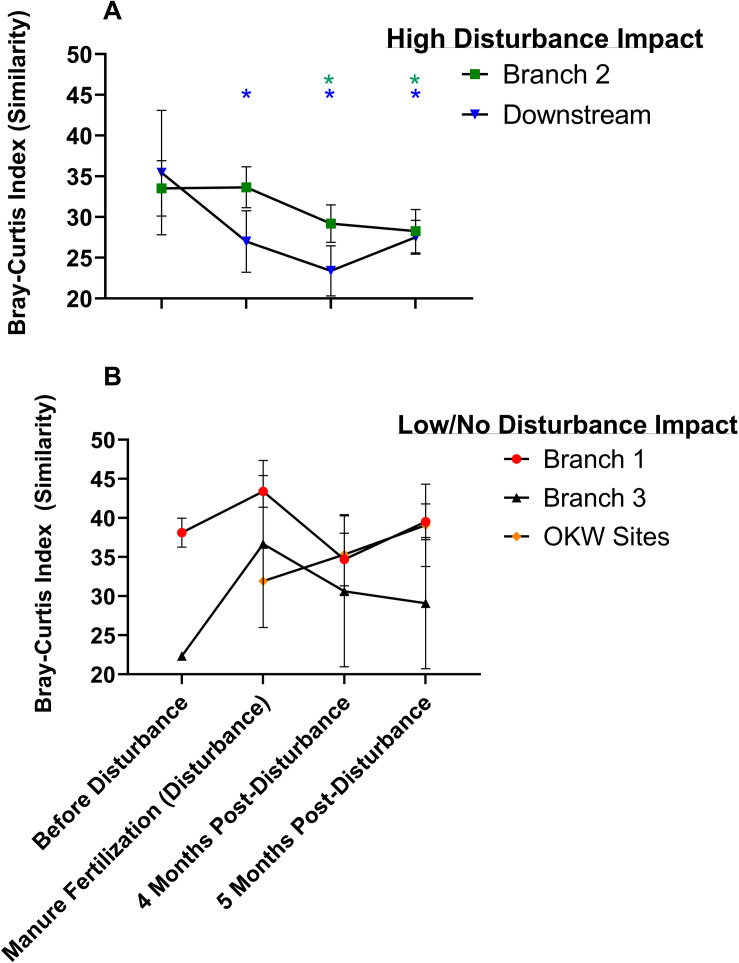
Changes in beta-diversity based on the mean ± standard deviation of Bray-Curtis Index (similarity) in **(A)** river branches receiving high disturbance impact and **(B)** river branches receiving low or no disturbance impact. Significant decreases (PERMANOVA and *post hoc* tests, *p* < 0.05) in microbial community composition similarity within river branches between the pre-disturbance and disturbance/post-disturbance states are indicated with * in colors corresponding to the sampling branch; only high disturbance river branches were significantly less similar between the pre and post-disturbance sampling timepoints.

The manure fertilization period in Kewaunee County begins in mid-April annually, serving as a repeated press disturbance to surrounding ecosystems. We hypothesized that sediment microbial communities of the highly impacted Kewaunee River watershed would be resilient to the annual manure fertilization disturbance because of the repeated, long-term exposure to microbial and chemical stressors in the region. We calculated the resistance and resilience of sediment microbial communities, focusing on the Kewaunee River watershed, based on alpha diversity (richness and evenness) and beta diversity (community composition). Within sample sediment microbial richness and evenness did not differ significantly pre- and post-disturbance at any sampling location (Supplementary Figures S1A,B); however, the within sample diversity at branches highly impacted by manure fertilization disturbance was higher by 4 months post-disturbance compared to the pre-disturbance state (Supplementary Figures S1C,D; Branch 2 and Downstream). A deeper investigation of the resistance and resilience of microbial community composition was estimated using PERMANOVA and ANOSIM statistics to determine if significant differences in the beta diversity of microbial communities both pre-and post-disturbance existed throughout both the entirety of the Kewaunee River watershed (temporal differences only; Table 1) and/or by river branch location (temporal and spatial differences; Figures 6A,B). At the watershed level, significant differences in the beta diversity of sediment microbial community composition between pre- and post-disturbance samples were identified, indicating microbial communities are not resilient up to 5 months after disturbance (Table 1). Although microbial communities within pre- and post-disturbance samples at the watershed level were significantly dissimilar (*p* < 0.01), microbial community composition of the post-disturbance samples were more similar to one another than samples pre- or during manure fertilization disturbance (*R* = −0.031, *p* > 0.05). This result suggests sediment microbial communities impacted by manure fertilization disturbance reach a new steady state by 5 months post-disturbance (Table 1).

As stated previously, the branches of the Kewaunee River watershed are differentially impacted by manure fertilization and agricultural runoff; thus, we analyzed if each individual river branch displayed the same temporal pattern and lack of resistance/resilience identified within the watershed as a whole. We discovered that the lack of resistance and resilience identified in Kewaunee River sediments was driven by only two of the four branches: the highly impacted Branch 2 and Downstream. The other two, less impacted branches of the Kewaunee River were not dissimilar over time and were instead resistant to microbial community compositional changes (Figure 6B). Additionally, we analyzed OKW sites which have no impact from CAFO farming for compositional differences pre- to post-disturbance and found that they were also not significantly different (Figure 6B). These results clearly indicate that the differences in microbial community composition found pre- and post- disturbance in this study are not simply due to seasonal temporal turnover or seasonal changes in environmental variables but rather can be attributed to manure fertilization disturbance because the temporal effects are only found in branches of the river highly impacted by manure fertilization runoff and agricultural activity. This strengthens support for our hypothesis of manure fertilization disturbance as the driver of changes in sediment microbial community composition at highly impacted river locations. Additionally, these results are supported by significant increases in the abundance of taxa associated with nutrients or pollutants from agricultural runoff at highly impacted locations (see Figure 4 above). Other studies have shown that microbial communities may take several years to exhibit resilience to disturbance (Enwall et al., 2007; Allison and Martiny, 2008; Zhang et al., 2017). In highly impacted areas of chronic contamination such as the Kewaunee River watershed, microbial communities may never return to a “pre-disturbance” state as they are continually exposed to multiple stressors from agricultural land runoff (microbial pathogens, heavy metals, nutrients, antibiotics, etc.).

One limitation of this study is that samples were collected over 1 year; thus, additional sample collection over multiple years is necessary to fully tease apart the relationship between disturbance, resilience, and seasonal temporal turnover in freshwater sediments. It is important to note that the temporal changes in microbial community composition associated with agricultural runoff disturbance in this study could also be associated with seasonal changes that occur in temperate regions such as Kewaunee County, WI. For example, the concentration of dissolved oxygen in surface waters is known to change seasonally with temperature (Rounds et al., 2013) but also with increased concentrations of organic pollutants (Graczyk and Sonzogni, 1991; Bricker et al., 2008). In this study, dissolved oxygen was shown to significantly impact variation in microbial community composition (Figure 4) and did change significantly during each sampling timepoint, with the highest measured concentrations in February and the lowest in September/October (data not shown). The Kewaunee River watershed, as a whole, displayed a temporal trend in microbial community composition; thus, without further investigation, one would be inclined to link seasonal changes in environmental variable concentration with the observed differences in microbial community composition. However, as we note above, the strong temporal trend was *only* identified in branches of the Kewaunee River that are highly impacted by intensive agriculture disturbance. This temporal trend disappears entirely in less impacted branches and the unimpacted OKW sites, supporting our hypothesis that agricultural runoff disturbance and associated spatial differences drive changes in microbial community composition in these sediments, not seasonal differences. These results highlight the complexity of analyzing the impacts of press disturbance in natural environments.

### Identified Potential Pathogenic Genera and Species in Freshwater Sediments Are Associated With the Cattle Microbiome

Using manure as cropland fertilizer introduces a number of human health risks into the environment including potential pathogenic bacteria (Oun et al., 2014). Due to the presence of 15 CAFO operations and manure fertilized cropland throughout the study area, we predicted that potential pathogens associated with the cattle microbiome would be detected more frequently in sediments at river locations impacted by agricultural runoff from intensive agriculture. Across all samples (including manure samples), 889 ASVs were mapped to a total of 46 genera known to contain human pathogens equating to 5.5% of the total sequence variants observed in this study (Supplementary Table S4). Of the 889 ASVs, 93 (10.5%) were classified to the species level using the strict 100% sequence matching method. Of these 93, 12 ASVs (12.9%) were identified as known human pathogen species equating to a total of nine species (Supplementary Table S4). We note that this method does not confirm pathogenicity of the identified species but does confirm their presence in these sediments.

Multiple potential human pathogens associated with the cattle microbiome were identified to the species level in both manure and sediment samples including *Acinetobacter lwoffii, Aeromonas sobria*, and *Arcobacter skirrowii* (Supplementary Table S4). Although *Acinetobacter, Aeromonas*, and *Arcobacter* genera are ubiquitous in freshwater environments, these genera contain opportunistic pathogens whose presence has been shown to increase following manure pollution (McAllister and Topp, 2012; Xiong et al., 2015). This result, together with the increased diversity of potential pathogens within the Kewaunee River watershed following the start of the manure fertilization period in late April, supports our hypothesis. We anticipated the detection of a diverse array of pathogens in Branch 2 and Downstream of the Kewaunee River but were surprised to also see an increase in pathogen diversity in Branch 1 due to the lack of CAFO farms impacting these locations (Supplementary Table S5). However, Branch 1 locations still receive agricultural runoff from manure fertilized cropland which demonstrates the widespread contamination in the county. Additionally, OKW locations which are unimpacted by intensive agriculture do not show a similar increase in pathogen diversity with time (Supplementary Table S5). While 16S rRNA sequence data alone cannot predict functional pathogenicity potential of the identified species, the presence of these species and changes in their abundance might be considered in human health risk assessments. Pathogen contamination of freshwater resources is estimated to impact more than 480,000 km of river ecosystems in the United States alone (Pandey et al., 2014), and manure is a primary source of these non-native bacteria in freshwater environments (Cabral, 2010). In Kewaunee County, both recreational freshwaters and groundwater drinking wells are at risk from manure runoff contamination due to the highly fractured karst bedrock geology. Thus, the identification and abundance of potential pathogens from agricultural sources should be further monitored in addition to the resistance and resilience of sediment microbial communities to infiltration by these potential pathogens.

## Conclusion

Sediment microbial communities are a core, but relatively understudied, component of freshwater ecosystems. Because sediments serve as sites of accumulation and transfer of pollutants and a reservoir of microorganisms (Devarajan et al., 2015), they are excellent indicators of long-term contamination from both point and NPS. Microbial communities within these sediments provide additional information about the health and functioning of the ecosystem as changes in core microbial taxa and functions can be reflective of environmental disturbance. In this study, we evaluated the impacts of long-term press disturbance on sediment microbial community composition and function in freshwater ecosystems. We found that sediment microbial communities within the highly impacted branches of the Kewaunee River are not resilient to manure fertilization disturbance over short timescales but do reach an alternative steady state. Sediment microbial community composition also varies significantly in association with local concentrations of agricultural pollutants. Microbial community functions in impacted freshwater sediments included anaerobic functions suggesting depleted dissolved oxygen and possible eutrophication events at locations highly impacted by manure runoff. Additionally, nine potential pathogens were identified to the species level, three of which are known to be associated with or originate from the cattle microbiome. Together, our results suggest agricultural land runoff from intensive livestock farming practices increases risks to environmental and human health by disseminating pollutants into the ecosystem and reducing the ability of the ecosystem to resist the influx of harmful bacteria and toxic chemicals.

This study does have a few limitations including the short timescale of the study and seasonal sampling. Additionally, calculating resistance and resilience under a natural press disturbance is difficult due to the continual contamination in the region and a lack of clearly defined “disturbance” timepoints. However, despite these limitations, the results presented here provide a strong case demonstrating the impact of press disturbance in freshwater sediments indicating microbial communities lose their potential to resist long-term, repeated stressors which alters ecosystem health and functioning.

This study highlights the impact of intensive agricultural contamination on the freshwater sediment microbial communities that serve as drivers of ecosystem processes. Further environmental monitoring is needed to determine the mechanism of specific biological and chemical drivers that contribute to decreased water quality and their impact on the resistance and resilience of freshwater microbial communities following pulse and press disturbances. Results from this study suggest that microbial communities exposed to repeated, long-term environmental manure contamination do not fully recover by five most post-disturbance, indicating that manure fertilization policies need to be re-evaluated to reduce exposure in surrounding ecosystems. Preventative measures, including construction of wetlands, buffer strips, and winter cover crop planting, aimed at reducing chemical and microbiological pollutants from manure runoff into freshwater sources should be implemented to protect environmental and human health.

## Data Availability Statement

Raw sequence data generated for this study were submitted to the NCBI Sequence Read Archive (SRA) under accession: PRJNA555250.

## Author Contributions

KH and RB designed the sampling regimen and identified collection locations. RB collected and processed samples and wrote the initial manuscript draft. RB and AB analyzed sequence data and performed statistical analysis. KH, AB, and SS edited and provided suggestions for the final version of the manuscript. All authors contributed to the article and approved the submitted version.

## Conflict of Interest

The authors declare that the research was conducted in the absence of any commercial or financial relationships that could be construed as a potential conflict of interest.
