# Supplementary material for: Freshwater Sediment Microbial Communities Are Not Resilient to Disturbance From Agricultural Land Runoff
Source: Front Microbiol. 2020 Oct 15;11:539921. doi: 10.3389/fmicb.2020.539921 (PMC7593329; doi:10.3389/fmicb.2020.539921)

***Supplementary Material***

| **Table S1.** GPS Coordinates and characteristics of sampling sites* within Kewaunee and Door counties, Wisconsin. Adapted from **Beattie *et al.* 2018.** | | | | |
| --- | --- | --- | --- | --- |
| ***Sampling Site Characteristics*** | | | | |
| *Site* | *Expanded Site Name* | *GPS Coordinates (Lat., Long.)* | *Site Clusters* | *Watershed* |
| KRS | Kewaunee River Source | 44.6180, -87.6024 | Source of KR, Branch 1 | Kewaunee |
| Maple | Casco Creek Maple | 44.6021, -87.6021 | Branch 1 | Kewaunee |
| Crevice | Casco Creek Crevice | 44.5837, -87.6022 | Branch 1 | Kewaunee |
| River | Kewaunee River River Road | 44.5743, -87.6846 | Branch 2 | Kewaunee |
| School | School Creek Valley Road | 44.5645, -87.6825 | Branch 2 | Kewaunee |
| KR54 | Kewaunee River County Road 54 | 44.5560, -87.6612 | Branch 2 | Kewaunee |
| Rockledge | Kewaunee River Rockledge | 44.5451, -87.6422 | Branch 2 | Kewaunee |
| Church | Casco Creek Church | 44.5559, -87.6180 | Branch 1 | Kewaunee |
| SCBig | Scarboro Creek | 44.5205, -87.6479 | Branch 3 | Kewaunee |
| SCKR | Scarboro Creek Kewaunee River | 44.5142, -87.6218 | Branch 3 | Kewaunee |
| KRCA | Kewaunee River County Road C-A | 44.5164, -87.6084 | Downstream | Kewaunee |
| BPKR | Bruemmer Park | 44.4606, -87.5580 | Downstream | Kewaunee |
| Loop | Kewaunee River Loop | 44.4779, -87.5271 | Downstream | Kewaunee |
| KRM | Kewaunee River Mouth | 44.4636, -87.5044 | Mouth of KR, Downstream | Kewaunee |
| DDUS | CAFO impacted stream | 44.6754, -87.5885 | DD | Ahnapee |
| DDDS | CAFO impacted stream | 44.6546, -87.5951 | DD | Ahnapee |
| OKW1 | Outside Kewaunee Watershed Site 1 | 44.8497, -87.4895 | OKW | Red River and Sturgeon Bay |
| OKW2 | Outside Kewaunee Watershed Site 2 | 44.8095, -87.5865 | OKW | Red River and Sturgeon Bay |
| OKW3 | Outside Kewaunee Watershed Site 3 | 44.7706, -87.6558 | OKW | Red River and Sturgeon Bay |

*Manure samples were also collected from a <200 head beef farm (May and September 2017) and a CAFO farm (September 2017) for a total of 3 samples.

| **Table S2.** Permuted multivariate analysis of variance results for comparisons of microbial community composition beta diversity based on Bray-Curtis dissimilarity. Bolded values indicate significant differences (*p*<0.05). | | |
| --- | --- | --- |
| **MANOVA** | | |
| *Variable* | *Pseudo-F* | *P(perm)* |
| Sampling Month | 1.9611 | **0.002** |
| Sample Watershed | 3.4723 | **0.001** |
| Sample Location nested in Sample Watershed | 3.9108 | **0.001** |
| **MANOVA Pairwise Tests- Watershed** | | |
| *Groups* | *t* | *P(perm)* |
| Kewaunee, Ahnapee | 1.6352 | **0.001** |
| Kewaunee, Red River and Sturgeon Bay | 1.642 | **0.003** |
| Ahnapee, Red River and Sturgeon Bay | 1.7482 | **0.001** |
| **MANOVA Pairwise Tests- Location within Kewaunee Watershed** | | |
| *Groups* | *t* | *P(perm)* |
| Downstream, B1 | 2.3095 | **0.001** |
| Downstream, B2 | 1.3878 | **0.008** |
| Downstream, B3 | 1.9258 | **0.002** |
| B1, B2 | 2.3057 | **0.001** |
| B1, B3 | 1.7388 | **0.005** |
| B2, B3 | 2.0373 | **0.001** |

| **Table S3.** DistLM marginal tests of the proportion of measured environmental variables influence on the variation in microbial community composition. Model variables include only those at or nearing statistical significance as determined by *P*. | | | |
| --- | --- | --- | --- |
| Variable | *Pseudo-F* | Proportion of variation explained | *P** |
| N | 1.5507 | 0.028958 | **0.037** |
| pH | 1.4943 | 0.027934 | 0.087 |
| DO | 2.1114 | 0.03902 | **0.006** |
| Coliforms | 1.9776 | 0.036637 | **0.011** |
| E_coli | 1.5577 | 0.029085 | **0.038** |
| Be | 2.283 | 0.042058 | **0.003** |
| Mg | 1.587 | 0.029615 | **0.036** |
| K | 3.2379 | 0.058618 | **0.001** |
| Ca | 1.5531 | 0.029001 | 0.073 |
| V | 2.3555 | 0.043335 | **0.002** |
| Fe | 1.5638 | 0.029195 | 0.07 |
| Co | 2.1926 | 0.040459 | **0.002** |
| Ni | 2.1757 | 0.04016 | **0.007** |
| As | 2.3797 | 0.043761 | **0.006** |
| Se | 1.7105 | 0.031846 | **0.025** |
| Mo | 2.0361 | 0.037681 | **0.007** |
| Ag | 2.1516 | 0.039732 | **0.003** |
| Ti | 1.8445 | 0.034257 | **0.013** |
| Pb | 1.5611 | 0.029147 | **0.042** |

***Bold** values indicate statistical significance.

| **Table S4.** Diversity of identified genera and species containing potential human pathogens in benthic sediments of northeastern Wisconsin. | |
| --- | --- |
| ***Acidaminococcus*** | ***Leptospira*** |
| ***Acinetobacter*** | ***Lysinibacillus*** |
| *Acinetobacter lwoffi* | ***Mycobacterium*** |
| ***Aerococcus*** | *Mycobacterium doricum* |
| ***Aeromonas*** | ***Mycoplasma*** |
| *Aeromonas sobria* | ***Myroides*** |
| ***Alloprevotella*** | ***Ochrobactrum*** |
| ***Arcobacter*** | ***Odoribacter*** |
| *Arcobacter cryaerophilus* | ***Paenibacillus*** |
| *Arcobacter skirrowii* | ***Pantoea*** |
| ***Bacillus*** | ***Parabacteroides*** |
| ***Bacteroides*** | ***Propionibacterium*** |
| ***Brevundimonas*** | ***Pseudomonas*** |
| ***Chryseobacterium*** | *Pseudomonas alcaligenes* |
| ***Comamonas*** | *Pseudomonas oryzihabitans* |
| ***Coxiella*** | ***Pseudonocardia*** |
| ***Delftia*** | ***Psychrobacter*** |
| ***Enterobacter*** | ***Ralstonia*** |
| ***Enterococcus*** | ***Rhodococcus*** |
| ***Erysipelothrix*** | *Rhodococcus rhodochrous* |
| ***Escherichia/Shigella*** | ***Rickettsia*** |
| *Escherichia/Shigella coli* | ***Serratia*** |
| ***Fibrobacter*** | ***Sphingomonas*** |
| ***Flavobacterium*** | ***Staphylococcus*** |
| ***Gordonia*** | ***Stenotrophomonas*** |
| ***Klebsiella*** | ***Sutterella*** |
| ***Legionella*** | ***Yersinia*** |
| ***Lelliottia*** |  |

| **Table S5.** Pathogens detected to the species level separated by month and river location. | | | | |
| --- | --- | --- | --- | --- |
|  | February | May | September | October |
| Branch 1 | *Mycobacterium doricum* | *Aeromonas sobria Pseudomonas alcaligenes* | *Acinetobacter lwoffli Aeromonas sobria Arcobacter cryaerophilus Escherichia coli/ Shigella coli Mycobacterium doricum* | *Acinetobacter lwoffli Aermonas sobria Arcobacter cryaerophilus Arcobacter skirrowii Mycobacterium doricum Pseudomonas alcaligenes Rhodococcus rhodochrous* |
| Branch 2 | *Aeromonas sobria Mycobacterium doricum Pseudomonas alcaligenes Rhodococcus rhodochrous* | *Acinetobacter lwoffli Aeromonas sobria Arcobacter cryaerophilus Arcobacter skirrowii Pseudomonas alcaligenes* | *Aeromonas sobria Pseudomonas oryzihabitans* | *Acinetobacter lwoffli Aermonas sobria Arcobacter cryaerophilus Pseudomonas alcaligenes* |
| Branch 3 | *Aeromonas sobria Arcobacter cryaerophilus Escherichia coli/Shigella coli Pseudomonas alcaligenes* | *Aeromonas sobria Escherichia coli/ Shigella coli* | *Aeromonas sobria Arcobacter cryaerophilus Arcobacter skirrowii* | *Aeromonas sobria* |
| Downstream | *Acinetobacter lwoffli Arcobacter cryaerophilus Mycobacterium doricum* | *Acinetobacter lwoffli Aeromonas sobria* | *Acinetobacter lwoffli Aeromonas sobria Pseudomonas alcaligenes* | *Aeromonas sobria Escherichia coli/ Shigella coli Mycobacterium doricum Pseudomonas alcaligenes* |
| OKW | *NA* | *Aermonas sobria* | *Aeromonas sobria Pseudomonas alcaligenes* | *Aeromonas sobria* |
| DD | *Aeromonas sobria Rhodococcus rhodochrous* | *Aeromonas sobira Pseudomonas alcaligenes* | *NA* | *Aeromonas sobria Pseudomonas alcaligenes* |
| Manure | *NA* | *Acinetobacter lwoffli Aeromonas sobria Arcobacter cryaerophilus Arcobacter skirrowii Rhodococcus rhodochrous* | *Acinetobacter lwoffli Arcobacter cryaerophilus Arcobacter skirrowii* | *NA* |

**Figure S1.** Microbial response to manure fertilization disturbance, estimated with changes in the mean ± standard error of alpha-diversity over time. All indices during disturbance, 4 months post-disturbance, and 5 months post-disturbance were compared to the pre-disturbance state within sampling location (individual samples clustered by river branch). Measured alpha diversity indices include: A) Chao1 Index, B) ACE Index, C) Shannon Diversity Index, and D) Inverse Simpson Index. Significant differences in alpha diversity within branch over time were compared using ANOVA followed by post-hoc tests (*p*<0.05). Only Branch 2 and Downstream branches of the Kewaunee River were significant different compared to the pre-disturbance state for Shannon Index and Inverse Simpson index (C-D); an added trend line and * signify the upward trend in diversity following disturbance at these two river branches.


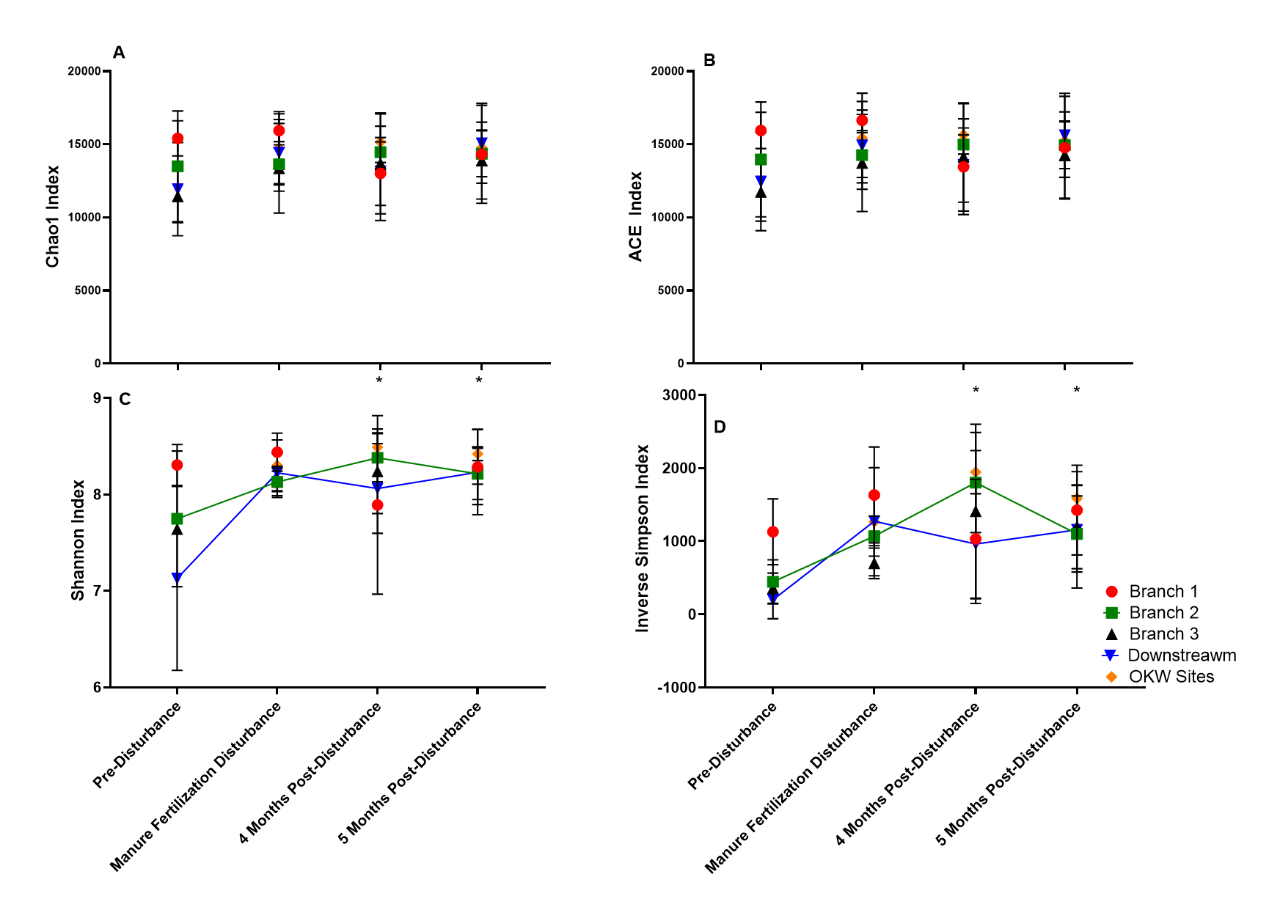

Supplement: Supplementary file 1 [file Data_Sheet_1.docx]
